# QuickStats

**Published:** 2013-08-23

**Authors:** LaJeana D. Howie

**Figure:**
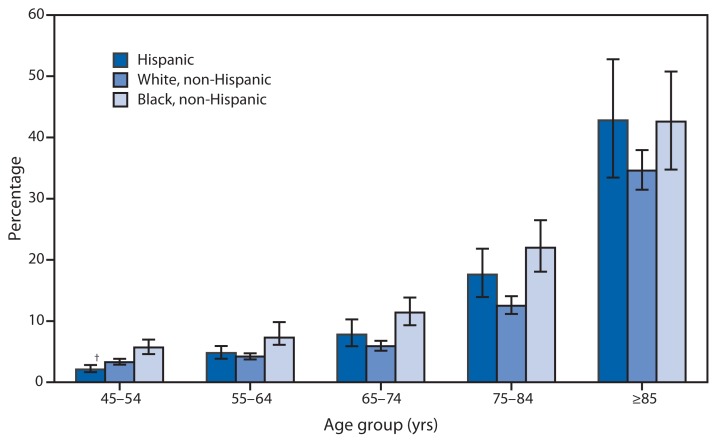
Percentage of Adults Aged ≥45 Years Who Need Help with Routine Activities* by Age Group and Selected Race/Ethnicity — National Health Interview Survey, United States, 2011 ^*^ Estimates are based on an affirmative response to the question, “Because of a physical, mental, or emotional problem, do you need the help of other persons in handling routine needs, such as everyday household chores, doing necessary business, shopping, or getting around for other purposes?” ^†^ 95% confidence interval.

Needing help with routine activities increased steadily with age for all racial/ethnic groups. Non-Hispanic blacks were more likely to need help with routine activities compared with Hispanics and non-Hispanic whites among those aged 45–74 years. Among adults aged 45–54 years, Hispanics were least likely to need help with routine activities. However, the pattern changes among adults aged ≥75 years; Hispanics and non-Hispanic blacks were both more likely to need help with routine activities than non-Hispanic whites.

**Sources:** CDC. National Health Interview Survey, 2011.

CDC. Health Data Interactive. Available at http://www.cdc.gov/nchs/hdi.htm.

